# Transcriptional profile reveals the physiological responses to prey availability in the mixotrophic chrysophyte *Poterioochromonas malhamensis*

**DOI:** 10.3389/fmicb.2023.1173541

**Published:** 2023-10-04

**Authors:** Mingyang Ma, Wentao Yang, Hong Chen, Wanwan Ke, Yingchun Gong, Qiang Hu

**Affiliations:** ^1^Institute for Advanced Study, Shenzhen University, Shenzhen, China; ^2^Department of Biochemistry and Molecular Medicine, Keck School of Medicine, University of Southern California, Los Angeles, CA, United States; ^3^Faculty of Synthetic Biology, Shenzhen Institute of Advanced Technology, Chinese Academy of Sciences, Shenzhen, China; ^4^CAS Key Laboratory of Quantitative Engineering Biology, Shenzhen Institute of Synthetic Biology, Shenzhen Institute of Advanced Technology, Chinese Academy of Sciences, Shenzhen, China; ^5^State Key Laboratory of Freshwater Ecology and Biotechnology, Institute of Hydrobiology, Chinese Academy of Sciences, Wuhan, China

**Keywords:** transcriptome, prey availability, feeding behavior, physiology, *Poterioochromonas malhamensis*

## Abstract

Mixotrophic flagellates, which have diverse nutritional modes and play important roles in connecting the microbial loop with the classical food chain, are ideal models to study the mechanisms of adaptation between different nutritional modes in protists. In their natural ecosystems, mixotrophic flagellates may encounter microalgal prey of different digestibility, which may affect the carbon flow. To date, a molecular biological view of the metabolic processes in the mixotrophic flagellate *Poterioochromonas malhamensis* during nutritional adaptation and feeding on microalgal prey of different digestibility is still lacking. Accordingly, this study focused on the gene expression differences in *P. malhamensis* under autotrophy, being fed by the digestible microalga *Chlorella sorokiniana* GT-1, and being fed by the indigestible microalga *C. sorokiniana* CMBB-146. Results showed that the growth rate of *P. malhamensis* under autotrophy was much lower than that when fed by digestible microalgae. Addition of *C. sorokiniana* CMBB-146 could only increase the growth rate of *P. malhamensis* in the first 3 days, but the cell concentration of *P. malhamensis* started to decrease gradually after 4 days. Compared to autotrophic *P. malhamensis*, total 6,583 and 3,510 genes were significantly and differentially expressed in *P. malhamensis* fed by digestible microalgae and indigestible microalgae, respectively. Compared to autotrophic cells, genes related to the ribosome, lysosome, glycolysis, gluconeogenesis, TCA cycle, β-oxidation, duplication, and β-1,3-glucan in *P. malhamensis* grazing on digestible prey were up-regulated, while genes related to light harvesting and key enzymes referring to chlorophyll were down-regulated. Genes related to apoptosis and necrosis in *P. malhamensis* were up-regulated after grazing on indigestible microalgae compared to the autotrophic group, which we suggest is associated with the up-regulation of genes related to lysosome enzymes. This study provides abundant information on the potential intracellular physiological responses of *P. malhamensis* during the process of nutritional adaptation.

## 1. Introduction

Mixotrophic flagellates are a group of protists that can obtain energy and carbon via photoautotrophy, chemoheterotrophy (also called osmotrophy), and phagotrophy (Jones, 2000; Burkholder et al., 2008). Over the last few decades, the important ecological roles played by these organisms have gradually been recognized. For instance, in some acidic lakes, as primary producers, mixotrophic flagellates can dominate the plankton (Sanders et al., 2000). On the other hand, they are important consumers of bacteria and phototrophic pico-phytoplankton in aquatic ecosystems (Mitra et al., 2014). Therefore, these organisms play a pivotal role in connecting the microbial loop with the classical food chain (Moorthi et al., 2009; Sanders, 2011). Furthermore, most mixotrophic flagellates are members of the chrysophyceae, dinophyceae, cryptophyceae, and dictyochophyceae families (Stoecker and Lavrentyev, 2018). Considering their diverse nutritional modes, these mixotrophic flagellates are ideal models to study the adaptation mechanisms of different nutritional modes in protists (Sanders, 2011).

*Poterioochromonas malhamensis* is a common mixotrophic flagellate that is widely distributed in aquatic ecosystems (Ma et al., 2023). The nutritional characteristics and relationships among different nutritional modes of *P. malhamensis* have been widely studied (Ma et al., 2023). It is considered to be a predominately heterotrophic mixotroph as photosynthesis contributes less than 7% of its total carbon budget in the simultaneous presence of abundant organics and light (Sanders et al., 1990). Under different nutritional conditions, the cell morphology, physiology, and biochemical composition of *P. malhamensis* will change greatly (Ma et al., 2022b). For example, the chloroplasts in *P. malhamensis* cells will be degraded and the chlorophyll-*a* content per cell will also decrease when organics are added into autotrophic cultures (Holen, 2011). The cell volume of *P. malhamensis* will increase after swallowing organic particles via food vacuoles or assimilating dissolved organics to form polysaccharide vacuoles (chrysolaminarin vesicles) (Ma et al., 2018, 2021). The protein and amino acid content of *P. malhamensis* will decrease when transforming from autotrophy to heterotrophy, while the carbohydrate and fatty acid contents show opposite trends (Ma et al., 2022b). Despite these findings, thus far, the molecular responses of *P. malhamensis* to different nutritional modes have rarely been studied (Ma et al., 2023). However, the rapid development of transcriptomic analysis has provided a high throughput approach that can be used to better understand the physiological and molecular responses of mixotrophic flagellates to different nutritional modes, or even to potentially identify the genes connected with specific nutritional strategies (Caron et al., 2017).

Among the different nutritional modes of *P. malhamensis*, phagotrophy has attracted the most attention. The prey spectrum of *P. malhamensis* is broad, including microalgae, bacteria, organic particles, and inorganic particles (Zhang and Watanabe, 1996). Numerous studies have shown that *P. malhamensis* has a strong grazing ability on *Microcystis* cells and can degrade microcystin efficiently (Ou et al., 2005; Zhang et al., 2010; Ma et al., 2022a). Therefore, it is considered to be a potential biocontrol method for cyanobacterial blooms. Recently, this mixotrophic flagellate was found to be one of the main risk factors in the microalgal industry owing to its strong grazing of commercial microalgal cells, especially *Chlorella* (Ma et al., 2017). However, to avoid being ingested or digested by protozoan predators, some prey microorganisms have evolved a range of resistance mechanisms (Martinez-Garcia et al., 2012; Gong et al., 2016). For instance, the presence of an S-layer in the cell wall of prey bacteria can inhibit the grazing ability of *Poterioochromonas* (Tarao et al., 2009). Meanwhile, we have also isolated a strain of *Chlorella sorokiniana* that can resist the predation by of *P. malhamensis* (Ma et al., 2019). However, the effect of grazing-resistant prey on the nutritional modes and gene expression of *P. malhamensis* is still not clear.

Accordingly, this study mainly focused on the gene expression differences of *P. malhamensis* under autotrophic conditions, fed with digestible microalgae, and fed with indigestible microalgae. To analyze the correlation patterns among genes across different nutritional conditions, we employed weighted gene co-expression network analysis (WGCNA), a widely used system biology method for gaining insights into genes from various metabolic pathways that exhibit similar expression patterns (Farhadian et al., 2021). To the best of our knowledge, this is the first study of the transcriptomic response of *P. malhamensis* to microalgal prey with different digestibility, the findings of which may provide insights into the mechanisms underlying the nutritional adaptation of this mixotrophic flagellate.

## 2. Materials and methods

### 2.1. Organisms and cultures

The predator, *P. malhamensis* CMBB-1, preserved in the China General Microbiological Culture Collection Center (No. 11620), was isolated from a crashed *Chlorella* culture. The digestible strain, *C. sorokiniana* GT-1 (GT-1), was isolated from South Lake in Guangzhou, China. The indigestible strain, *C. sorokiniana* CMBB-146 (CMBB-146), was isolated from the accidentally contaminated culture of *P. malhamensis*. *Poterioochromonas malhamensis*, was preserved in 250 mL flasks with AF-6 medium (Kato, 1982), while the two strains of *C. sorokiniana* were both cultivated using BG-11 medium (Rippka et al., 1979). The temperature of the culture room was 22°C–25°C, with a continuous illumination condition of 30–50 μmol photons m^−2^ s^−1^.

### 2.2. Cultivation of *Poterioochromonas malhamensis* under different nutritional conditions

The mixotrophic flagellate *P. malhamensis* was cultivated under three different nutritional conditions, including autotrophy, mixotrophy fed with the digestible strain GT-1, and mixotrophy fed with the indigestible strain CMBB-146. For mixotrophic growth, *P. malhamensis* was cultivated in BG-11 medium containing GT-1 or CMBB-146 at a concentration of 1.0 × 10^7^ cells mL^−1^. To eliminate the influence of culture medium, BG-11 medium was also used for autotrophic *P. malhamensis* culture. The initial concentrations of *P. malhamensis* in the different groups were all 3.0 × 10^5^ cells mL^−1^. Furthermore, to exhibit the difference in digestibility between GT-1 and CMBB-146, pure cultures of two strains of *C. sorokiniana* without addition of the predator were also set up. The initial concentrations of the two strains of *C. sorokiniana* in the control groups were both 1.0 × 10^7^ cells mL^−1^. The culture system consisted of glass column photobioreactors (φ, 5 cm) with a 700 mL working volume of medium. The medium in the column was continuously bubbled with CO_2_/air comprising 1–3% CO_2_ (*v*/*v*). The cultures were continuously illuminated with a light intensity of 100 μmol photons m^−2^ s^−1^ under a temperature of 22 ± 1°C. Each treatment had three replicates. The population dynamics of *P. malhamensis* and *C. sorokiniana* were monitored daily during a whole cultivation time of 10 days. Cell concentrations of *P. malhamensis* and *C. sorokiniana*, which was stained with 1% Lugol’s iodine, were counted using a hemocytometer at 400 × magnification. The morphologies of *P. malhamensis* cells under different nutritional conditions were recorded using microscopy (BX53, Olympus, Japan). The ultrastructures of the two strains of *C. sorokiniana* were observed using transmission electron microscopy according to the method described in Ma et al. (2018).

### 2.3. Inhibition effect of *Chlorella sorokiniana* culture filtrates on the feeding ability of *Poterioochromonas malhamensis*

Four different culture filtrates (including a pure culture of GT-1, pure culture of CMBB-146, co-culture of GT-1 and *P. malhamensis*, and co-culture of CMBB-146 and *P. malhamensis*) collected from different culture periods (day 1, day 3, and 7), were used to test their inhibition effects on the feeding ability of *P. malhamensis*. These filtrates were obtained through filtering the culture medium through a filter membrane with a pore size of 0.22 μm. The feeding ability of *P. malhamensis* was evaluated based on the clearance rate of thermally killed GT-1 cells. Prey cells grown at logarithmic phase were killed by heating at 75°C for 5 min. For each treatment, the same amounts of the predator, *P. malhamensis* (initial concentration of 2.0 × 10^5^ cells mL^−1^), and dead prey, *C. sorokiniana* (initial concentration of 2.0 × 10^7^ cells mL^−1^), were co-cultivated with different filtrates. The cell concentrations of predator and prey were counted as described in section 2.2. The culture system consisted of 100 mL flasks with a 50 mL working volume of BG-11. Each treatment had three replicates. The clearance rate (*n*, %) was calculated as *n* = (*N*_0_ − *N*_t_) / *N*_0_ × 100%, where *N*_0_ and *N*_t_ denote the prey concentrations in the initial stage and end stage, respectively. The culture period was 36 h. The temperature of the culture room was 22°C–25°C, with a continuous illumination condition of 30–50 μmol photons m^−2^ s^−1^. The differences in clearance rate among the different groups were tested using one-way analysis of variance followed by Tukey’s honestly significant difference test.

### 2.4. RNA extraction, complementary DNA library construction, and transcriptome sequencing

The *P. malhamensis* cells after cultivation or co-cultivation for 2 days were sampled for transcriptome analysis. Aliquots of cultures (100 mL) were harvested by centrifuging at 1500 g for 5 min. The cell pellet was immediately transferred to liquid nitrogen to stabilize profiles of gene expression. Total RNA was extracted with an RNeasy Mini Kit (Qiagen, Hilden, Germany) according to the manufacturer’s instructions. The quality and quantity of RNA were both assessed with a NanoDrop 8,000 spectrophotometer (Thermo Scientific, USA) and electrophoresis on a 1% agarose gel. The construction of complementary DNA libraries and the subsequent sequencing were executed by NextOmics Bioscience Co., Ltd. (Wuhan, China). Sequencing was performed on the Illumina Hiseq X10 platform.

### 2.5. RNA-seq data analysis

Raw data derived from RNA-seq were assessed using FastQC (Andrews, 2010). Low-quality reads, including adapter sequences and reads with a quality score lower than 20, and ambiguous bases, were removed using Trim Galore v0.6.4 (Krueger, 2012). Trimmed reads with lengths less than 20 bp were filtered and the remaining reads with lengths more than 20 bp were then aligned to the *P. malhamensis* CMBB-1 genome by using Tophat (Trapnell et al., 2009) with default settings. Transcripts Per Million (TPM) were used to quantify the expression of transcripts. To compare similarity across samples, TPM were used for principal component analysis using the prcomp function in R. Hierarchical clustering was performed to visualize the Pearson correlation coefficient between any two samples. The number of reads was counted using featureCount (Liao et al., 2014) at gene level. A matrix including RNA counts of each gene in each sample was imported in the DESeq2 package (Love et al., 2014), where library size normalization and logarithmic transformation were performed to detect significant differentially expressed genes with an adjusted *p*-value threshold of 0.05 as well as absolute log-fold change larger than 2.

### 2.6. Functional enrichment analysis of different expression genes (DEGs)

To evaluate the functional enrichment of significant up−/down-regulated genes in the biological process, gene ontology (GO) analysis was performed using BinGO (Maere et al., 2005). Kyoto Encyclopedia of Genes and Genomes (KEGG) pathway enrichment analysis was carried out in the clusterProfiler (Yu et al., 2012) package with default settings. Hypergeometric testing was applied to identify over-representation terms for DEGs against the whole genome as the background with Benjamini and Hochberg correction for multiple testing. Corrected *p-*values <0.05 were reported as significant terms and the significantly enriched terms were plotted as bubbles with the ggplot2 package.

### 2.7. Gene co-expression network construction and functional annotation

All DEGs were loaded into the WGCNA package (Langfelder and Horvath, 2008) for constructing the co-expression network by following the manual protocol provided by this package. In brief, the pickSoft threshold function was applied to choose the appropriate power parameter for network construction by applying the scale-free topology criterion. Then, we employed the function TOMsimilarity to compute the Pearson correlations between all pairs of genes. After that, an adjacent matrix generated corresponding to the interconnections between any two genes was subjected to hierarchical clustering analysis. Clusters were identified on the dendrogram using the cutreeHybrid function embedded in the dynamicTreeCut package (Langfelder et al., 2008), wherein a cluster corresponds to a module, which consists of a group of genes with high topological overlap. For each module, a module eigengene was defined as the first principal of the expression matrix to represent the weighted average expression profile using the function moduleEigengenes. Finally, the module membership was determined by calculating the correlation between the gene expression values and the module eigengene under the default threshold.

### 2.8. Identification of significant modules and hub genes

Associations of modules and phenotypic traits were measured as the Pearson correlation between the trait and the module eigengene expression profile. For genes in highly positive correlated modules to traits, GO enrichment analysis was conducted as previously described. Briefly, significantly enriched terms were defined by the hypergeometric test with a threshold of false discovery rate less than 0.05 through comparing the genes in a module with all the genes as the background. Moreover, we calculated the degree of centrality to indicate the number of links for each gene in the highly correlated modules using the Cytoscape (Shannon et al., 2003) plugin networkanalyzer (Assenov et al., 2008). Genes ranked in the top 20 in terms of their degree of centrality values were considered as the hub genes for further analysis and validation.

## 3. Results

### 3.1. Community dynamics of *Poterioochromonas malhamensis* and *Chlorella sorokiniana* under different environmental conditions

**Figure 1:**
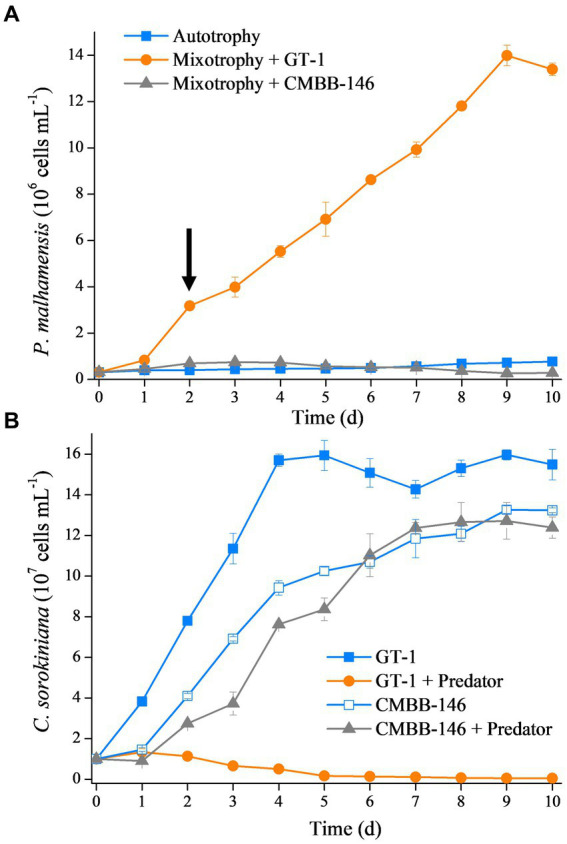
**(A)** Growth curves of *P. malhamensis* without prey (autotrophy), fed with digestible prey *C. sorokiniana* GT-1 (mixotrophy + GT-1), and fed with indigestible prey *C. sorokiniana* CMBB-146 (mixotrophy + CMBB-146). The arrow denotes the sampling time for transcriptome, i.e., day 2. **(B)** Growth curves of *C. sorokiniana* GT-1 without predator (GT-1), *C. sorokiniana* GT-1 with predator *P. malhamensis* (GT-1 + predator), *C. sorokiniana* CMBB-146 without predator (CMBB-146), and *C. sorokiniana* CMBB-146 with predator *P. malhamensis* (CMBB-146 + predator). Standard deviation was represented by error bars, some of which were too small to be visible. *n* = 3.

The growth rate of *P. malhamensis* under different environmental conditions varied largely. As shown in Figure 1A, the cell concentration of autotrophic *P. malhamensis* increased gradually but at a very slow speed. Addition of *C. sorokiniana* CMBB-146 (hereafter, simply CMBB-146) could only increase the growth rate of *P. malhamensis* in the first 3 days, but the cell concentration of *P. malhamensis* started to decrease gradually after 4 days. The final concentration of *P. malhamensis* feeding on CMBB-146 on day 10 was only 2.8 × 10^5^ cells mL^−1^, which was lower than that of the initial cell concentration (3.0 × 10^5^ cells mL^−1^) and the autotrophy group on day 10 (7.7 × 10^5^ cells mL^−1^). The growth rate of *P. malhamensis* cultivated with *C. sorokiniana* GT-1 (hereafter, simply GT-1) as prey was the highest, and the final concentration on day 10 reached 1.3 × 10^7^ cells mL^−1^. The cell ultrastructures of GT-1 and CMBB-146 were similar except for their cell walls (Supplementary Figures S1A,B). The cell wall of GT-1 was adhered with a cell membrane in a loose form, while a tight form was apparent for CMBB-146. It was found, based the microscopic observation, that *P. malhamensis* was able to ingest either GT-1 or CMBB-146 but preferred to ingest more GT-1 than CMBB-146. Meanwhile, the chloroplasts of *P. malhamensis* ingesting GT-1 were less intact and distinct than those of autotrophic *P. malhamensis* or mixotrophic *P. malhamensis* fed with CMBB-146 (Supplementary Figures S1C–E).

From the perspective of prey, the addition of *P. malhamensis* as a predator greatly affected the growth of GT-1 but had little impact on the growth of CMBB-146 (Figure 1B). The maximum cell concentration of GT-1 in the control group reached 1.6 × 10^8^ cells mL^−1^ on day 5 from an initial cell concentration of 1.0 × 10^6^ cells mL^−1^, whereas GT-1 in the treatment group ceased growth from day 1 and the cell concentration gradually decreased to 5.2 × 10^5^ cells mL^−1^. For CMBB-146, the cell concentration in the co-culture group was lower than in the control group in the first 5 days, whereas it was similar in the two groups after 6 days. These results indicate that GT-1 is a digestible prey for P. malhamensis, while CMBB-146 is indigestible prey for *P. malhamensis*.

### 3.2. Inhibition effect of *Chlorella sorokiniana* culture filtrates on the feeding ability of *Poterioochromonas malhamensis*

**Figure 2:**
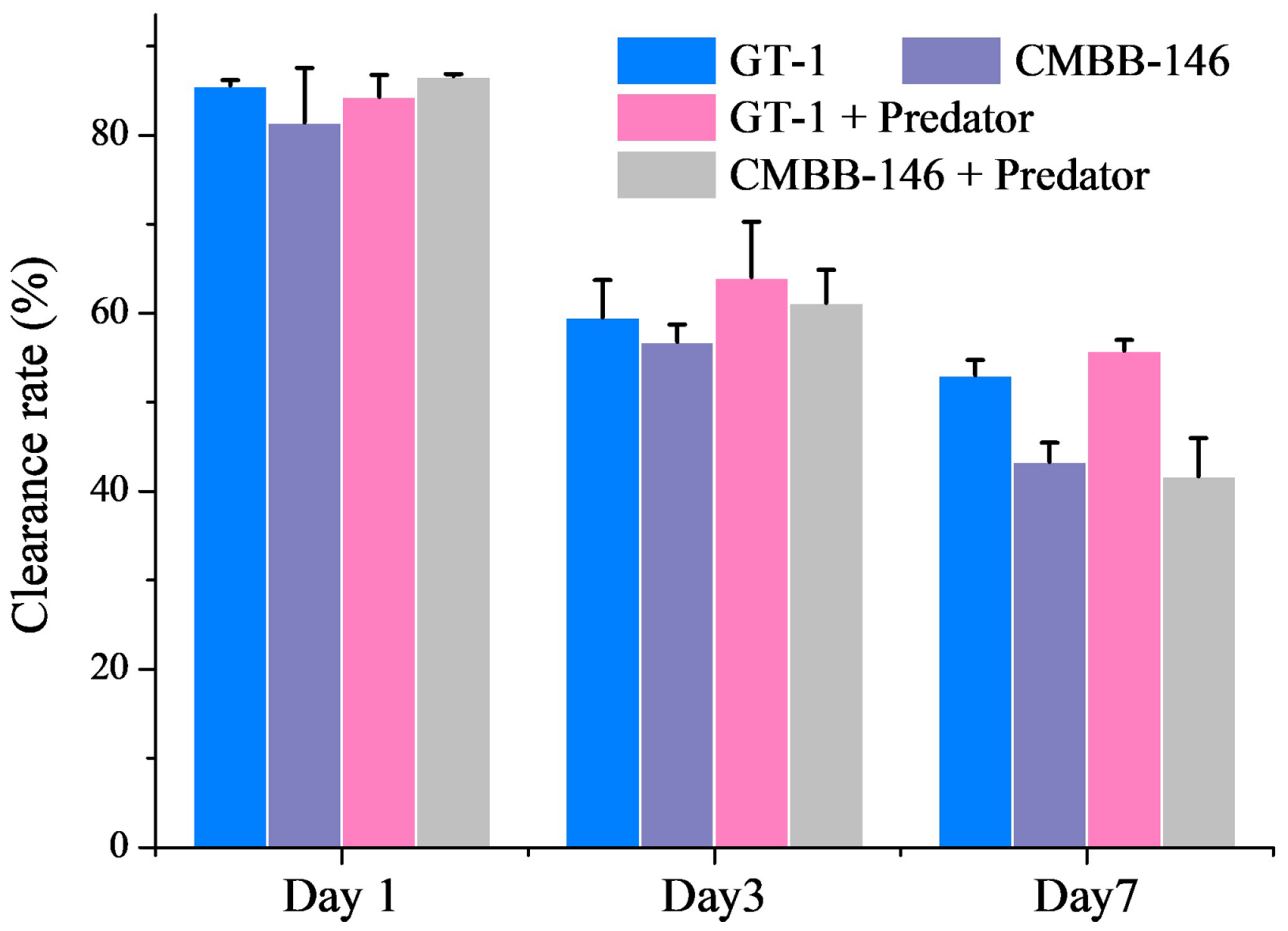
Effect of culture medium filtrates collected from different treatment groups and different culture times on the feeding ability of *P. malhamensis*. ‘GT-1’ and ‘CMBB-146’ mean that the culture medium filtrate was collected from the pure culture of *C. sorokiniana* GT-1 and *C. sorokiniana* CMBB-146, respectively. ‘GT-1 + Predator’ and ‘CMBB-146 + Predator’ mean that the culture medium filtrate was collected from the co-culture of *P. malhamensis* – *C. sorokiniana* GT-1 and *P. malhamensis* – *C. sorokiniana* CMBB-146, respectively. The feeding ability of *P. malhamensis* was determined by calculating the clearance rate, which was based on the loss rate of thermally inactivated GT-1 cells consumed by *P. malhamensis* within 36 h. Error bars represent the standard deviation. *n* = 3.

On the whole, the clearance rates of *P. malhamensis* on prey in different culture filtrates collected on the same culture day were similar (Figure 2). However, the clearance rate of *P. malhamensis* on prey in the filtrates from the same culture all decreased with time. The clearance rates of *P. malhamensis* on prey in the filtrates from day 1 and day 3 were all around 80.0 and 60.0%, respectively. The clearance rates of *P. malhamensis* on prey in the filtrated culture collected from ‘GT-1’ and ‘GT-1 + predator’ on day 7 were 53.0 and 55.8%, respectively, which were slightly higher (*p* < 0.05) than those of ‘CMBB-146’ (43.3%) and ‘CMBB-146 + predator’ (41.7%). This suggested that the low feeding ability of *P. malhamensis* on CMBB-146 was not caused by releasing certain chemical inhibitors produced by CMBB-146 into the culture medium.

### 3.3. Transcriptome profiles of *Poterioochromonas malhamensis* under different nutritional conditions using bulk RNA-seq

**Figure 3:**
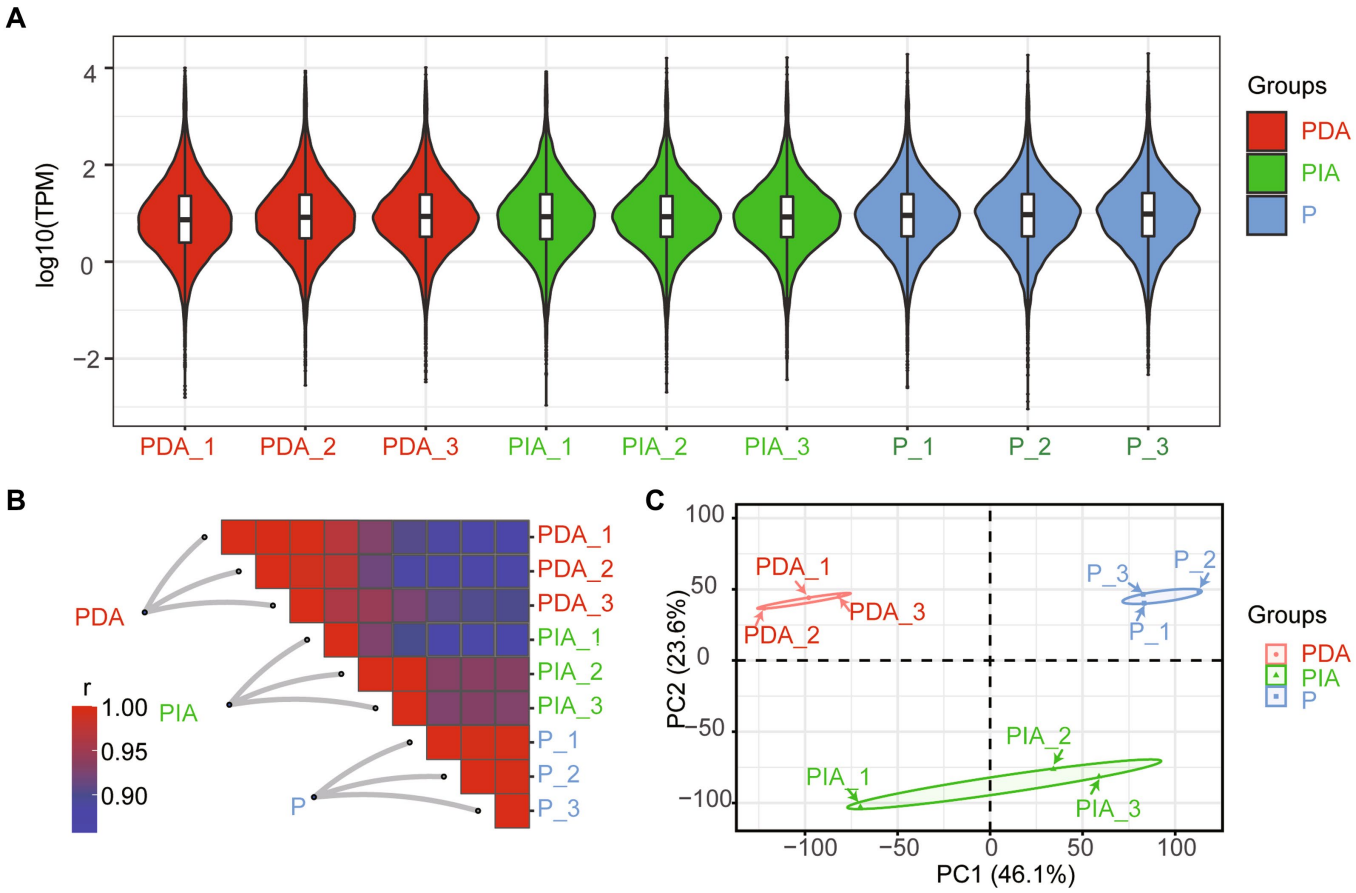
Overview of RNA-seq data under the mixed cultivation of *P. malhamensis* with two microalgae. **(A)** Violin plot showing the distribution of log10 transformed TPM mapped reads for each sample. **(B)** Heatmap of the Pearson’s correlation coefficient matrix between any two samples. **(C)** PCA plot for the transcriptome of *P. malhamensis* with or without microalgae mixed culture. P, *P. malhamensis* under autotrophy; PDA, *P. malhamensis* fed with digestible prey *C. sorokiniana* GT-1; PIA, *P. malhamensis* fed with indigestible prey *C. sorokiniana* CMBB-146.

To characterize the gene expression dynamics of *P. malhamensis* under different environmental conditions, we performed bulk RNA-seq in three states (autotrophy, hereafter referred to as P; mixotrophy with one digestible strain (GT-1), hereafter referred to as PDA; and mixotrophy with one indigestible strain (CMBB-146), hereafter referred to as PIA). As shown in Supplementary Table S1, a total of over 404.7 million clean reads were obtained, with the read number per library ranging from 37.3 to 57.9 million. About 90.4–97.5% of the reads per library were successfully mapped to the reference genome, with a unique mapping rate of 73.7–79.2% and a multiple mapping rate of 16.7–18.5% (Supplementary Table S1). The distributions of log2 (FPKM) were highly consistent (Figure 3A), suggesting a general uniformity and limited bias in the sequencing coverage among all the nutritional conditions. Moreover, hierarchical clustering showed that the samples in three biological replicates had a high level of correlation, while there was a relatively low degree of correlation among samples in different nutritional conditions (Figure 3B). A principal component analysis was conducted to further assess the quality of the RNA-seq data. As depicted in Figure 3C, distinct clusters were observed for each treatment group, with the exception of PIA_1, which diverged from the other replicates but still within an acceptable range. These results indicate that the observed differences in transcriptome gene expression were indeed attributable to the different nutritional conditions.

### 3.4. Distinct gene expression patterns under different nutritional conditions

**Figure 4:**
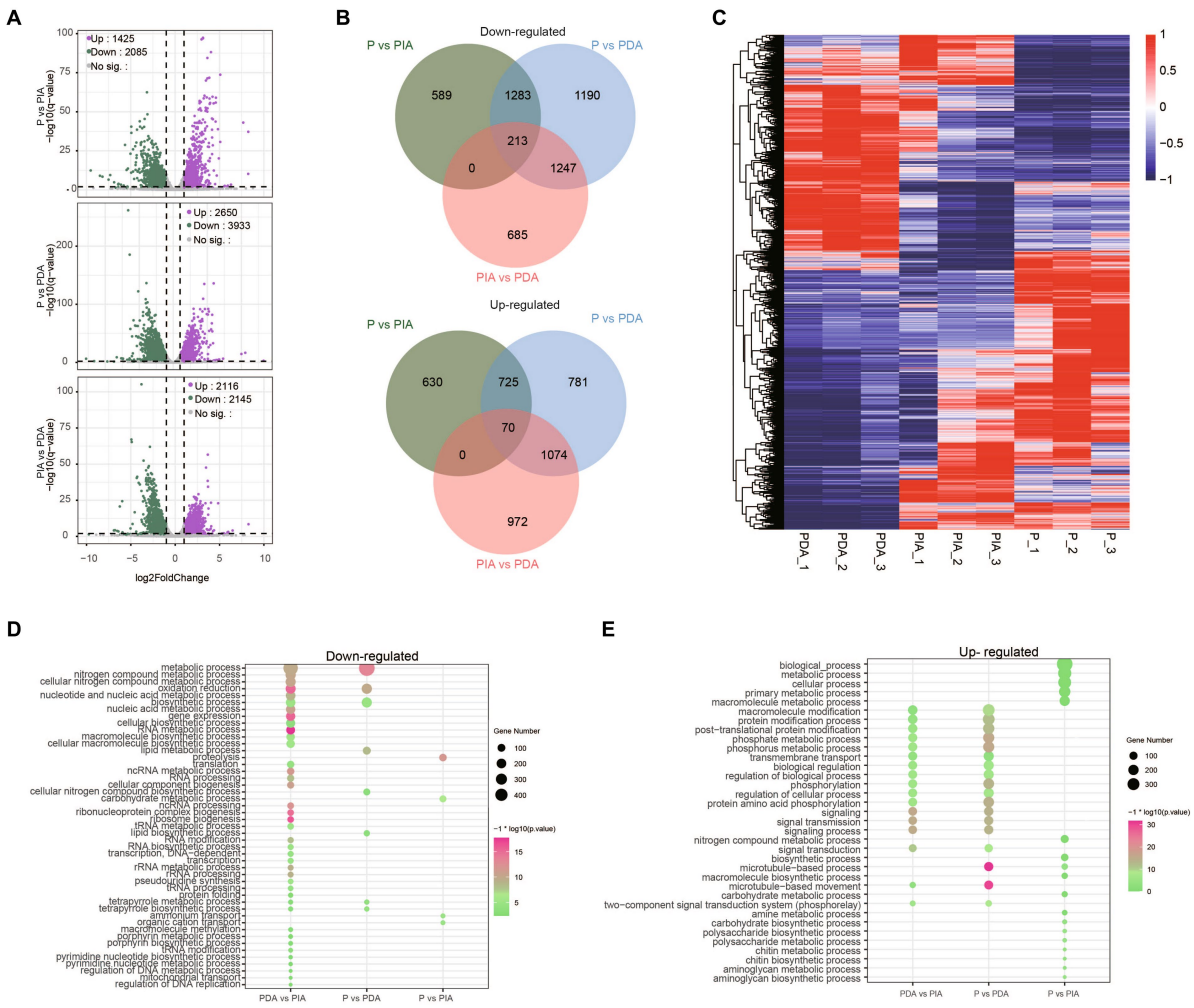
Characteristics of the *P. malhamensis* gene expression changes between any two treatments. **(A)** Volcano plots showing the fold change (*x*-axis) of normalized counts and adjusted *p*-value (*y*-axis) on the log scale of control versus indigestible microalgae (upper), control versus digestible microalgae (middle), and indigestible versus digestible (lower). Colored dots represent the significantly up-regulated genes (in purple), down-regulated genes (in green), and unaltered genes (in gray). **(B)** Venn diagrams displaying the number of up- and down-regulated genes that were shared or were specific to each group. **(C)** Hierarchical clustering analysis performed using the TPM on all the DEGs. Color coding represents the normalized expression value using a *z*-score, in which blue indicates low expression and red indicates high expression. The dendrograms on the left of the heat map show the hierarchical clustering of DEGs. **(D,E)** The enriched biological process terms for down-regulated genes and up-regulated genes, respectively. Dot size represents the gene count in enriched terms and the color scale indicates the significance level. P, *P. malhamensis* under autotrophy; PDA, *P. malhamensis* fed with digestible prey *C. sorokiniana* GT-1; PIA, *P. malhamensis* fed with indigestible prey *C. sorokiniana* CMBB-146.

We next conducted a differential expression analysis to identify genes related to the nutritional adaptation. Compared to P, 1425 up-regulated and 2085 down-regulated genes were identified in PIA, whereas almost 1.5 times more up-regulated (2650) and down-regulated genes (3933) were discovered in PDA (Figure 4A), indicating that more dramatic transcriptional changes occurred in the digestible group. Simultaneously, we also assessed the DEGs between PIA and PDA. A total of 4,261 genes were identified, of which 2,116 and 2,145 were significantly up-regulated and down-regulated, respectively (Figure 4A). To comprehensively explore the relationships of the DEGs among these three nutritional conditions, we examined the overlapping genes between each pair. The Venn diagram of up-regulated and down-regulated genes in PDA shows that more than 50% of DEGs were shared with PIA. Intriguingly, there were 2,437 up-regulated and 1855 down-regulated genes, accounting for 62.0 and 70.0% of the corresponding DEGs, respectively, identified to be differentially expressed exclusively in PIA (Figure 4B). However, we only identified 70 up-regulated and 213 down-regulated genes shared among the three conditions (Figure 4B), suggesting extremely significant changes in dynamic expression. In addition, all the DEGs were profiled by hierarchical clustering to show the expression patterns (Figure 4C). Our analysis revealed that the expression profiles of P and PDA exhibit an inverse relationship, with PIA displaying an intermediate expression pattern.

To gain insights into the distinct gene expression patterns during the adaptation of nutritional modes, we performed GO enrichment analysis to investigate the biological processes of the DEGs. In comparison of P vs. PDA, the down-regulated genes were significantly enriched in GO terms related to metabolic and biosynthetic processes, such as lipid metabolism, lipid biosynthesis, tetrapyrrole metabolism, and tetrapyrrole biosynthesis (Figure 4D), whereas the up-regulated genes were significantly enriched in GO terms related to signal transduction and macromolecule modification, including post-translational protein modification, phosphorylation, and the two-component signal transduction system (Figure 4E). Intriguingly, the enriched GO terms of down-regulated genes in P vs. PIA were involved in ammonium transport, organic cation transport, carbohydrate metabolism, and proteolysis process (Figure 4D); whereas, for the up-regulated genes, biological processes involved in nitrogen compound metabolism, chitin biosynthesis, and aminoglycan biosynthesis (Figure 4E) were significantly enhanced. Surprisingly, the enriched terms for the up-regulated genes were almost identical to those in PDA vs. PIA (Figure 4E), whereas the down-regulated genes with the most abundant GO terms were enriched in some unique pathways, such as tRNA modification, regulation of DNA replication, and mitochondrial transport (Figure 4D).

### 3.5. Distinct pathways inferred from KEGG under different nutritional conditions

**Figure 5:**
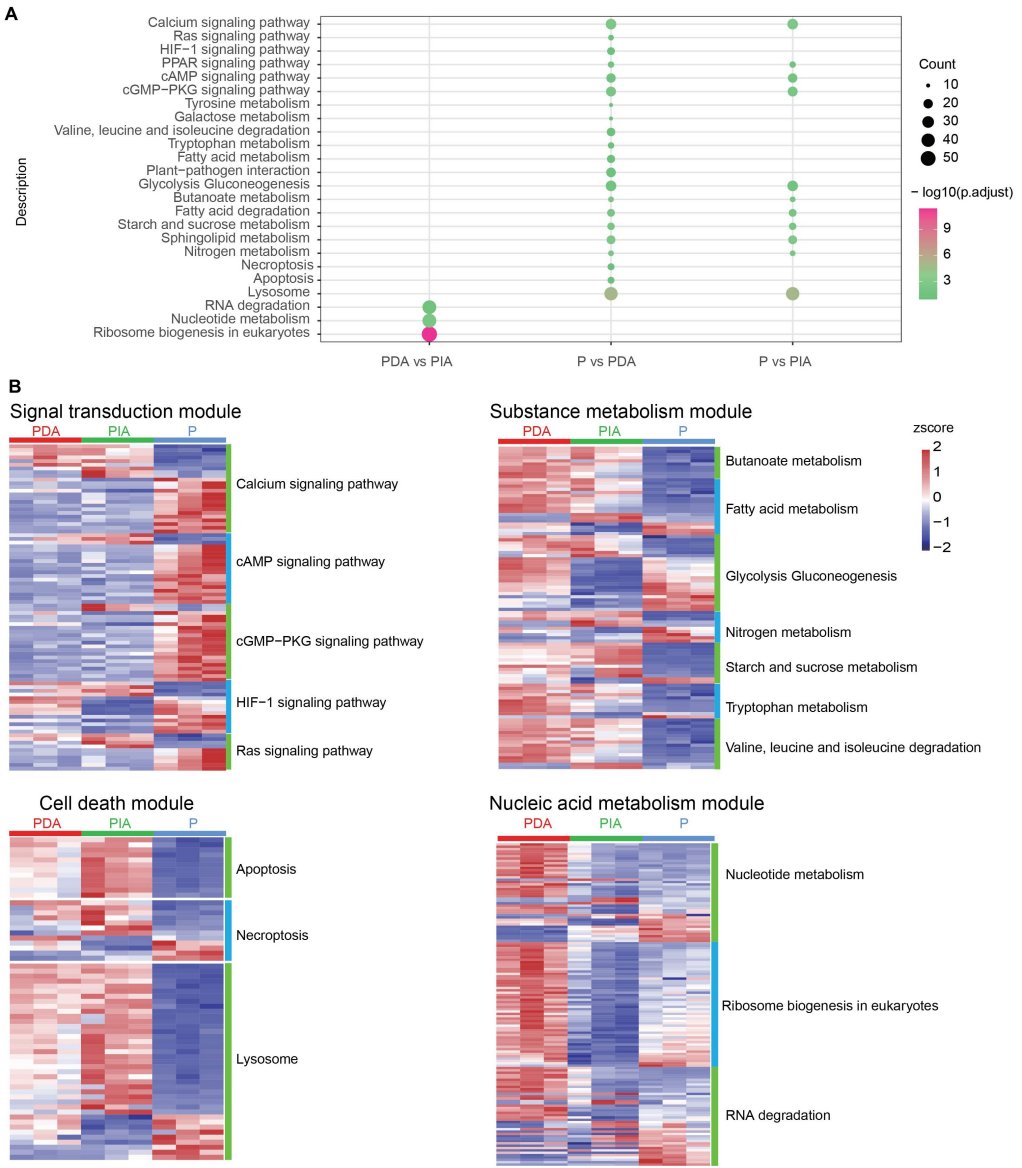
KEGG enrichment analysis and visualization of gene expression profile for DEGs. **(A)** The enriched KEGG pathways of DEGs. The dot size represents the gene count and the color scale represents the adjusted *p*-value. **(B)** Heatmaps showing gene expression changes in several enriched major pathways. The color scale represents the *z*-score normalized gene expression level (red, high expression; blue, low expression). P, *P. malhamensis* under autotrophy; PDA, *P. malhamensis* fed with digestible prey *C. sorokiniana* GT-1; PIA, *P. malhamensis* fed with indigestible prey *C. sorokiniana* CMBB-146.

Given that a diverse range of functions was enriched by up−/down-regulated genes, we investigated whether DEGs (including both up- and down-regulated genes) were differentially enriched at the pathway level. To this end, DEGs detected in each group were loaded into the clusterProfiler package for KEGG pathway enrichment analysis. According to the KEGG enrichment results, P vs. PDA and P vs. PIA showed overlap in some signaling pathways, including calcium signaling, cyclic adenosine monophosphate (cAMP) signaling, and cGMP-PKG signaling, as well as metabolic pathways such as glycolysis gluconeogenesis, nitrogen metabolism, and fatty acid degradation (Figure 5A). We also observed that genes differentially expressed in P vs. PDA and P vs. PIA were most significantly involved in the lysosome pathway. In contrast to P vs. PIA, there were several specific enriched pathways in P vs. PDA, such as signaling pathways related to Ras and hypoxia-inducible factor 1 (HIF-1), pathways related to amino acid metabolism, cell death related to necroptosis, and apoptosis (Figure 5A). In addition, we enriched pathways for DEGs in PDA vs. PIA. Genes involved in RNA degradation, nucleotide metabolism, and ribosome biogenesis showed significant enrichment in PDA vs. PIA.

To compare the transcriptional patterns in individual pathways, we grouped the pathways into four modules according to their functional characteristics: signal transduction, substance metabolism, cell death–related, and nucleic acid metabolism pathways (Figure 5B). Signal transduction comprised 88 DEGs, including 24 calcium signaling genes, 19 cAMP signaling genes, 21 cGMP-PKG signaling genes, 14 HIF-1 signaling genes, and 10 Ras signaling genes. Among these, 66 DEGs showed higher expression in the autotrophic mode (P) compared to the mixotrophic mode (Figure 5B; Supplementary Table S2). In the metabolism module, a total of 7 enriched pathways correspond to 102 DEGs were identified, including genes in butanoate metabolism, fatty acid metabolism, glycolysis gluconeogenesis, nitrogen metabolism, starch and sucrose metabolism, tryptophan metabolism, and valine degradation. When compared to P, 72 genes showed higher expression and 30 genes were down-regulated in both PIA and PDA. Interestingly, we observed that 8 genes involved in glycolysis gluconeogenesis, which controls the breakdown and production of glucose, were highly expressed in both PDA and P compared to PIA (Figure 5B; Supplementary Table S3). Cell death–related pathways, including 12 apoptosis related genes, 12 necroptosis related gene, and 39 lysosome related genes, showed that more than 50.0% genes with the highest expression in PIA (Figure 5B; Supplementary Table S4). For the nucleic acid module, we identified 42 nucleotide metabolism genes, 42 RNA degradation genes and 53 ribosome biogenesis genes. Almost all the genes in ribosome biogenesis pathway showed the highest expression in PDA, followed by P and then PIA, indicating their essential roles in DNA replication and RNA production to support protein synthesis during cell proliferation. Most of these genes in the nucleotide metabolism and RNA degradation were up-regulated in the PIA in compared with PDA, whereas some genes were down-regulated in compared with P (Figure 5B; Supplementary Table S5).

Our analysis also focuses on the expression levels of genes associated with the photosynthesis process and β-1,3-glucan metabolism. We found that many key genes associated with pigment synthesis, e.g., magnesium chelatase, ferrochelatase, and zeaxanthin epoxidase, were downregulated in PDA compared to P (Supplementary Table S6). Compared to P, the expression level of β-1,3-glucan synthase was higher in PDA, while the gene encoding β-1,3-glucanase exhibited opposite trends (Supplementary Table S6).

### 3.6. Gene co-expression networks under different nutritional conditions

**Figure 6:**
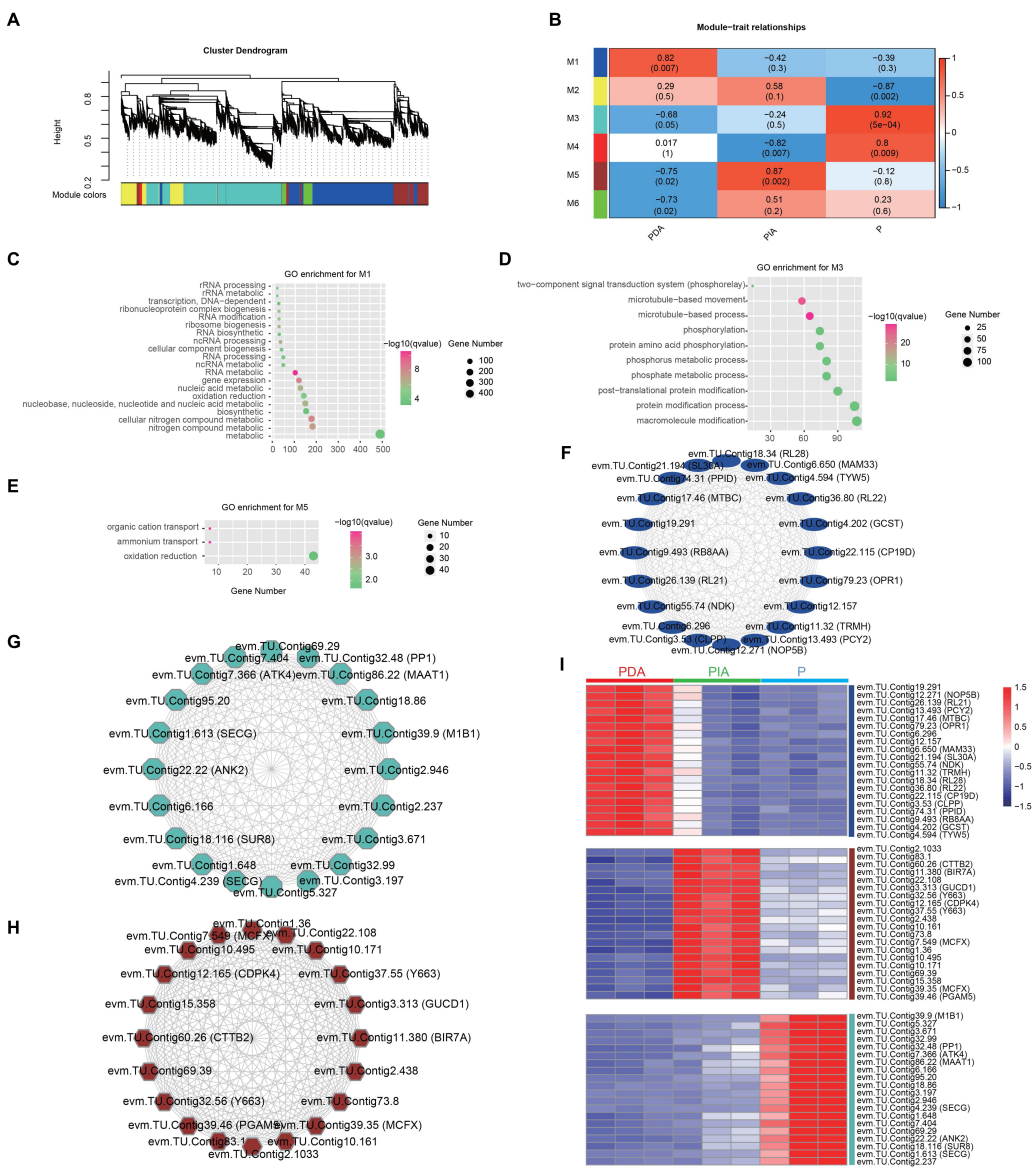
Co-expression gene modules identified by weighted gene co-expression network analysis. **(A)** Dendrograms of all DEGs clustered into five distinct modules based on a dissimilarity of consensus topological overlap measure. The colored row below the dendrogram shows the modules determined by the dynamic tree cut. Each color represents a module consisting of a group of highly connected genes. **(B)** Heatmap plot showing the relationships between the module and the trait weight. Each row and column correspond to the module eigengene and the trait weight, respectively. Red coloring represents positive correlation, while blue represents negative correlation. **(C–E)** GO enrichment analysis for genes in module 1 (M1, **C**), module 3 (M3, **D**) and module 5 (M5, **E**). **(F–H)** The top 20 hug genes in module 1 **(F)**, module 3 **(G)** and module 5 **(H)**. **(I)** Expression profile of the top 20 hub genes in module 1, 3 and 5. Red: high expression; blue: low expression. P, *P. malhamensis* under autotrophy; PDA, *P. malhamensis* fed with digestible prey *C. sorokiniana* GT-1; PIA, *P. malhamensis* fed with indigestible prey *C. sorokiniana* CMBB-146.

To understand the regulatory network underpinning the transition between nutritional modes, we constructed weighted gene co-expression networks based on all the DEGs among the three nutritional conditions. We identified a total of six co-expression modules (M1–M6), as indicated by different colors in the hierarchical tree. The gene numbers in the modules ranged from 62 to 3,316 (Figure 6A; Supplementary Table S7). We then correlated the six module eigengenes with the three nutritional conditions to investigate the significance of each module. The most significant correlations were found between M1 and PDA (*r* = 0.82, *p* = 0.007), M5 and PIA (*r* = 0.87, *p* = 0.002), and M3 and P (*r* = 0.92, *p* = 0.0005) (Figure 6B). Therefore, we speculate that genes involved in M1, M3 and M5 might be essential for the transition between feeding modes. GO enrichment analysis revealed that genes involved in M1 were involved in nucleic acid metabolism, including RNA modification, ncRNA processing, and rRNA processing (Figure 6C). The top 10 enriched terms in M3 were associated with the two-component signal transduction system, microtubule-based processes, and protein amino acid phosphorylation (Figure 6D). It is interesting to note that only three terms—related to organic cation transport, ammonium transport, and oxidation reduction—were highly enriched in M5 (Figure 6E). Collectively, the enriched terms in each module were consistent with the previous analysis of the distinct gene expression patterns and pathways in multiple nutritional modes, suggesting that the gene co-expression networks represented by the modules reveal functional implications.

By calculating the degree of centrality, which reflects the interactions between genes, we obtained the most highly connected hub genes in M1, M3 and M5 to further determine the key genes involved in nutritional adaptation. The top 20 hub genes in M1, including those encoding for 50S ribosomal protein L21 (RL21), 50S ribosomal protein L22 (RL22), and tRNA wybutosine-synthesizing protein 5 (TYW5), were related to RNA processing (Figure 6F). Consistent with the GO-enriched terms in M3, we also found genes involved in protein post-translational modifications, such as serine/threonine-protein phosphatase PP1 (PP1) and E3 ubiquitin-protein ligase MIB1 (MIB1) (Figure 6G). Additionally, genes including those encoding for calcium-dependent protein kinase 4 (CDPK4), responsible for the signal transduction process, putative ammonium transporter MTH_663 (Y663), and mitochondrial substrate carrier family protein X (MCFX), involved in substrate transport and energy supply, were also identified (Figure 6H). Notably, the highly specific expression patterns of these hub genes in individual modules indeed confirmed the functional significance (Figure 6I; Supplementary Table S8). The aforementioned results suggest that *P. malhamensis* may regulate the expression of genes belonging to different modules in response to different nutritional conditions.

## 4. Discussion

*Poterioochromonas malhamensis* has long been used as a model to study the feeding behavior and nutritional adaptation of mixotrophic flagellates. However, most related studies have focused on the population dynamics and physiological and biochemical responses of this mixotrophic flagellate under different environmental conditions (Lewitus and Caron, 1991; Zhang et al., 2009; Weisse and Moser, 2020). Our study compared the different gene expression profiles of *P. malhamensis* under autotrophy and mixotrophy (including feeding on digestible prey and indigestible prey), which aimed to provide in-depth information on the intracellular physiological responses of *P. malhamensis* during the process of nutritional adaptation and feeding on prey with different availability.

### 4.1. The expression of genes related to autotrophy and mixotrophy

The CO_2_ concentrating mechanism (CCM) is an important mechanism for phytoplankton to support high rates of photosynthetic carbon fixation and photosynthesis at low concentrations of CO_2_ in aquatic environments (Giordano et al., 2005). The CCM of chrysophytes has not been well studied, whereas previous studies have shown that most freshwater chrysophyte species have no CCM and rely on CO_2_ diffusion (Reinfelder, 2011). Therefore, it is generally assumed that the low photosynthetic rate of chrysophytes is due to the lack of a CCM. However, the transcriptome data in this study showed that *P. malhamensis* possess genes encoding proteins related to a CCM, such as carbonic anhydrase (CA) (Supplementary Table S6), which has also been observed in *Ochromonas* sp. CCMP1393 (Lie et al., 2018). It is noteworthy that the presence of CA alone does not mean the occurrence of a CCM in *P. malhamensis* cells, as these enzymes have other cellular functions. The expressed abundance of CA in autotrophic *P. malhamensis* was lower than that in mixotrophic *P. malhamensis* (Supplementary Table S6). Therefore, we suggest that CA in *P. malhamensis* cells only plays a role in transporting out the excessive CO_2_ generated by the high respiratory efficiency under mixotrophy and lack the activity of concentrating exogenous CO_2_.

The feeding behavior of *P. malhamensis* initiates a digestion process that produces metabolites, which subsequently trigger a cascade of intracellular physiological responses, including alterations in central carbon and nitrogen metabolism as well as photosynthesis. It has been widely demonstrated that the pigment content of *P. malhamensis* cells (e.g., chlorophyll-*a* and fucoxanthin) decrease obviously in the presence of exogenous prey or dissolved organics (Holen, 2011; Ma et al., 2022b). Correspondingly, the photosynthetic capacity of *P. malhamensis* also decreases when switching from autotrophy to heterotrophy or mixotrophy. However, compared with autotrophic cells, *P. malhamensis* cells in PDA upregulated most genes related to pigment synthesis (Supplementary Table S6), except for the key genes in these pathways, e.g., those encoding for magnesium chelatase in the chlorophyll synthesis pathway (Tanaka and Tanaka, 2007), ferrochelatase in the phycobillin synthesis pathway (Shimizu and Masuda, 2021), and zeaxanthin epoxidase in the fucoxanthin synthesis pathway (Wang et al., 2021). It was found that by reducing the expression of key enzymes associated with pigment synthesis, mixotrophic *P. malhamensis* cells reduced their pigment content.

Contrary to our expectations, there was upregulation of almost all genes related to photosynthesis, including Photosystem II, cytochrome b6/f, and ATP synthase, in mixotrophic *P. malhamensis* compared to autotrophic cells (Supplementary Table S6). However, the genes associated with the light-harvesting chlorophyll protein complex showed opposite trends (Supplementary Table S6), which has also been observed in another mixotrophic flagellate, *Ochromonas* sp. strain BG-1 (Lie et al., 2017). Therefore, it is hypothesized that *P. malhamensis* reduces the efficiency of photosynthesis by decreasing the light harvesting capacity in the presence of microalgal prey. Furthermore, we hypothesize that the decrease in light harvesting capability and efficiency of the photosynthetic machinery under mixotrophy may serve to reduce photo-oxidative stress and the excessive production of reducing agent (NADPH) in *P. malhamensis* cells when energy and carbon are readily available through prey digestion. Under mixotrophic conditions, intense photosynthetic activities will presumably lead to the accumulation of excess reducing agent in *P. malhamensis* cells, as the reductant would be generated from both the phagotrophic reactions and light reaction of photosynthesis.

Compared to autotrophic cells, mixotrophic *P. malhamensis* feeding on digestible prey exhibited up-regulation of carbon metabolism–related genes involved in glycolysis, gluconeogenesis, the TCA cycle, β-oxidation, and oxidative phosphorylation (Figure 5B), suggesting stronger glycolytic activity and more energy production during the metabolism of microalgal carbon sources under digestible microalgal treatment. This observation is consistent with previous findings for the mixotrophic alga *Ochromonas* sp. strain BG-1 (Lie et al., 2017) and the mixotrophic haptophyte *Prymnesium parvum* (Liu et al., 2015). A previous study also showed that phagotrophy contributed more than 90% of the total carbon budget of *P. malhamensis* under mixotrophic conditions (Sanders et al., 1990). Another study, which used the nanoSIMS method, documented that 89–99% of cellular carbon in the mixotrophic alga *Ochromonas* sp. strain BG-1 was derived from grazed prey (Terrado et al., 2017). Phagotrophy presumably also provides an abundant nitrogen source for mixotrophic flagellates because of the nitrogen-rich nature of prey. Large quantities of amino acids and glutamate are likely produced from the breakdown of prey’s proteins, and glutamate dehydrogenase produces ammonium during the conversion of glutamate to oxoglutarate (Lie et al., 2017). The expression of most ammonium transporter genes in *P. malhamensis* was up-regulated in the presence of digestible microalgae, which was also observed in mixotrophic *Ochromonas* sp. CCMP1393 (Lie et al., 2018). The up-regulated gene expression of glutamate dehydrogenase in the presence of digestible microalgae is consistent with the documented release of ammonium in different species of *Ochromonas* when these algae are grazing on prey (Lie et al., 2017).

β-1,3-glucan, commonly present in the form of chrysolaminarin vacuoles, is the major storage substance in chrysophytes (Myklestad and Granum, 2009). A previous study showed that *P. malhamensis* cells accumulated more than 50% of their β-1,3-glucan in dry weight through absorbing and assimilating enough glucose under dark conditions (Ma et al., 2021). Compared to autotrophic cells, mixotrophic *P. malhamensis* could also accumulate more β-1,3-glucan through up-regulating the gene encoding for β-1,3-glucan synthase and down-regulating the gene encoding for β-1,3-glucanase (Supplementary Table S6).

**Figure 7:**
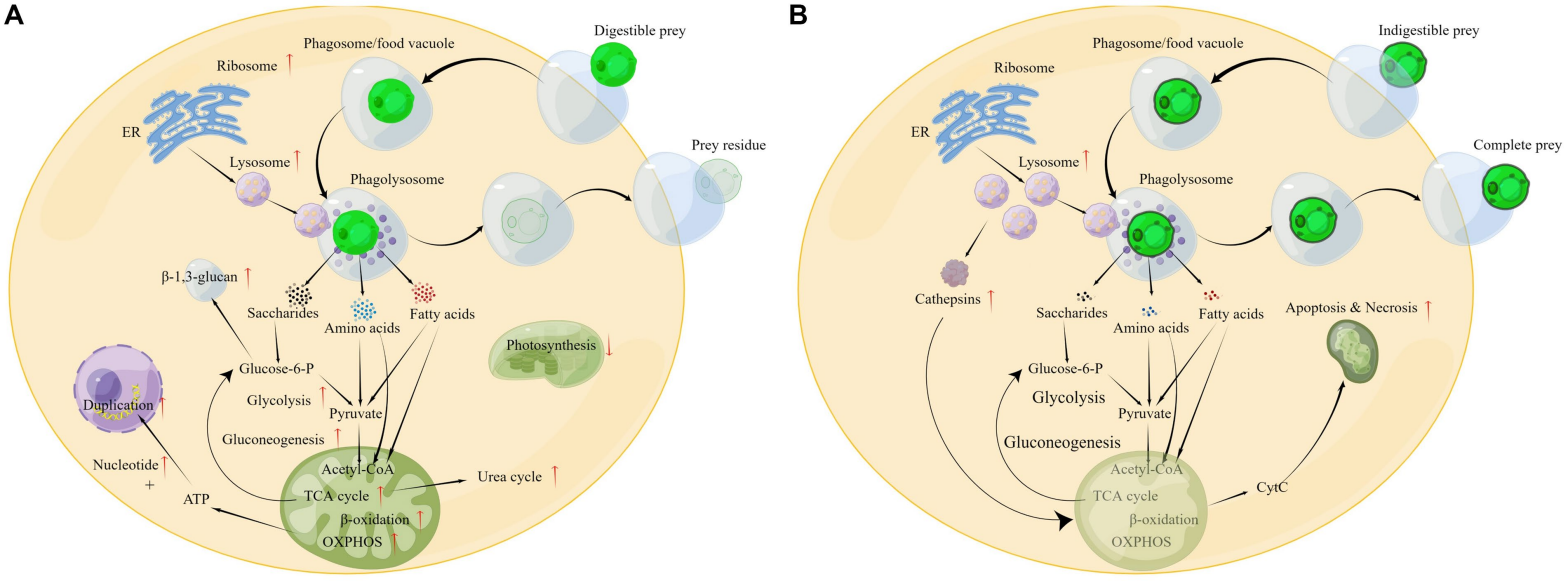
Different physiological responses of *P. malhamensis* when grazing on microalgae with different grazing resistance: **(A)** grazing on digestible microalgae; **(B)** grazing on indigestible microalgae. The pathway diagram was produced using the online software Figdraw.

On the basis of the above analysis, a global change in the cellular metabolic pathways of *P. malhamensis* when feeding on microalgae or other prey can be outlined as shown in Figure 7A. After the prey is engulfed in the phagosome (or food vacuole), the ribosome will synthesize various digestive enzymes and these enzymes will be delivered into the lysosome. The lysosome soon merges with the phagosome to form a phagolysosome, where the swallowed prey is digested. The digestive products, e.g., saccharides, ammonic acid, and fatty acids, accelerate the central carbon metabolism and nitrogen metabolism, as well as the rate of reproduction. Meanwhile, the photosynthetic efficiency is weakened, as the digestion products can provide abundant material and energy. After digesting the grazed microalgae, the prey residue is excreted from the *P. malhamensis* cells.

### 4.2. Physiological responses of *Poterioochromonas malhamensis* grazing on indigestible microalgae

Allelopathy is a common strategy for microalgae to resist grazing by predators, e.g., dimethylsulphoniopropionate secreted by the alga *Emiliania huxleyi* against the predator *Oxyrrhis marina* (Wolfe et al., 1997). Our results showed that the structural defense of the microalgal cell, rather than a specific allelopathic substance secreted by the microalgae, was responsible for the inability of *P. malhamensis* to feed on CMBB-146 (Figure 2). *Poterioochromonas malhamensis* is generally considered to be a predator that ingests prey non-selectively. It can ingest microalgae, bacteria, yeast, grain detritus, lipid droplets, and inorganic particles (Sanders and Porter, 1988). Furthermore, cannibalism has also been observed in *P. malhamensis* (Zhang and Watanabe, 1996). However, it expels the ingested prey that cannot be digested (Dubowsky, 1974). Therefore, selective digestion is considered to be the main mechanism responsible for the differential processing of prey particles in *P. malhamensis*. A previous study indicated that CMBB-146 could not be digested by *P. malhamensis* owing to its different cell wall composition compared to other digestible *Chlorella* species (Ma et al., 2019). However, during the initial period of co-culture, a large number of CMBB-146 cells were ingested by the predator, impacting the reproductive capacity of CMBB-146 (Ma et al., 2019). Consequently, a significant difference in the cell concentration of CMBB-146 between the control and experimental group was observed within the first 5 days (Figure 1B). The transcriptome data in this study provided more information on the intracellular physiological responses of *P. malhamensis* grazing on indigestible prey. Similar to *P. malhamensis* grazing on digestible prey, there was also an up-regulation of genes related to lysosome enzymes when *P. malhamensis* grazed on indigestible prey (Figure 5B). The result is consistent with a previous study in which the digestive enzyme activity of *P. malhamensis* was present in all different particle-containing vacuoles, including nutritive and non-nutritive particles (Dubowsky, 1974). These results indicate that the digestive enzyme activity in *P. malhamensis* cells is due to the presence of particulate material in the food vacuole and has nothing to do with the nature of the particle.

The cell concentration of *P. malhamensis* gradually decreased when grazing on indigestible microalgae, which was also observed in a previous study (Ma et al., 2019). Although the pathways of apoptosis and necrosis were only enriched in PDA, many genes related to these pathways in PIA were also up-regulated (Figure 5B). Furthermore, the expression abundance of some genes related to apoptosis and necrosis in PIA was higher than that of PDA (Supplementary Table S4). Therefore, according to these transcriptome data, the death of *P. malhamensis* might be caused by the up-regulation of genes related to apoptosis and necrosis after grazing on indigestible microalgae. Caspases, a family of cysteine proteases that are usually involved in apoptosis (Hyman and Yuan, 2012), were not found in the transcriptome data. It is worth noting that the expression of many lysosome enzyme genes in *P. malhamensis* grazing on indigestible prey was higher than on digestible prey (Supplementary Table S4). A previous study showed clearly that cathepsins can be released into the cytosol and initiate the lysosomal pathway of apoptosis through the cleavage of Bid and the degradation of anti-apoptotic Bcl-2 homologs after destabilization of the lysosomal membrane (Repnik et al., 2012). The released hydrolases further destabilize the mitochondrion membrane and result in the release of apoptosis factors, e.g., cytochrome C (cyt C). The released cyt C then activates caspase-9 and induces apoptosis (Wang, 2001). Meanwhile, the released hydrolases induced by the lysosomal membrane’s destabilization also initiate cell necrosis. Therefore, we propose the following possible physiological response mechanism of *P. malhamensis* during its grazing on indigestible prey (Figure 7B). A number of lysosomal enzymes are synthesized and transported into the lysosome, and the lysosome merges with the phagosome/food vacuole containing the indigestible microalgae to form a phagolysosome. As fewer nutrient products, e.g., saccharides, ammonic acid, and fatty acids, are obtained in the phagolysosome, more lysosome enzymes will be synthesized and transported into the phagolysosome. Following the egestion of indigestible microalgae with intact cell morphology by *P. malhamensis* cells, excess lysosomal enzymes may be released into the cytosol, leading to the destabilization of mitochondrial membranes and subsequent release of cytochrome c. This, in turn, may initiate the processes of apoptosis and necrosis.

### 4.3. Signal regulation during the nutritional adaptation between autotrophy and mixotrophy

The nutritional adaptation mechanism of mixotrophic flagellates is a topic that has long attracted the attention of evolutionists and protozoologists. Although the effect of exogenous dissolved organics or particulate prey on the chloroplast content and β-1,3-glucan content of *P. malhamensis* has been explored (Ma et al., 2022b), the molecular mechanism remains unknown. Our transcriptome data provide some preliminary clues toward understanding the signaling pathway that regulates the transformation between autotrophy and mixotrophy in *P. malhamensis* cells. Previous studies have documented that the addition of glucose to the culture medium will cause *P. malhamensis* to release its intracellular cAMP into the medium, while depletion of glucose in the culture medium by *P. malhamensis* will result in the accumulation of intracellular cAMP, as well as the formation of chlorophyll (Bressan et al., 1980; Handa et al., 1981). Therefore, the formation and release of cAMP is considered to be an important regulatory signal associated with the concentration of exogenous organics (e.g., glucose) and the onset of chlorophyll synthesis in *P. malhamensis*. Adenylate cyclase (AC) is the key enzyme that catalyzes the production of cAMP from ATP. It integrates positive and negative signals acting through G protein–coupled receptors (GPCRs) with other extracellular stimuli to precisely regulate the concentration of intracellular cAMP (Gehring, 2010). In addition, AC activity is also regulated by the calcium-calmodulin (Ca^2+^-CAM) signaling pathway. In our study, compared to autotrophic cells, the expression of genes related to inactivating AC (e.g., cyclic nucleotide gated channel subunit beta 1, calmodulin, guanine nucleotide-binding protein G (i/t) subunit alpha) was consistently down-regulated in mixotrophic *P. malhamensis* (Supplementary Table S9).

A high concentration of cAMP is associated with a depletion of glucose in both bacteria and animals (Tomkins, 1975). A high cAMP concentration can activate protein kinase, causing inactivation of glycogen synthetase and activation of phosphorylase, which accelerates the degradation of glycogen. With sufficient glucose, glycogen synthetase will be activated while phosphorylase will be inactivated (Robinson et al., 1968). Based on our transcriptome data, β-1,3-glucan showed similar trends to glycogen in synthesis and degradation when *P. malhamensis* lacked exogenous organics (equivalent to transforming from mixotrophy to autotrophy), i.e., enzyme synthesis decreased and enzyme degradation increased. Since β-1,3-glucan and glycogen both play a role in the storage of substances in organisms, the synthesis and degradation of β-1,3-glucan in *P. malhamensis* should be regulated by the same pathway as glycogen.

**Figure 8:**
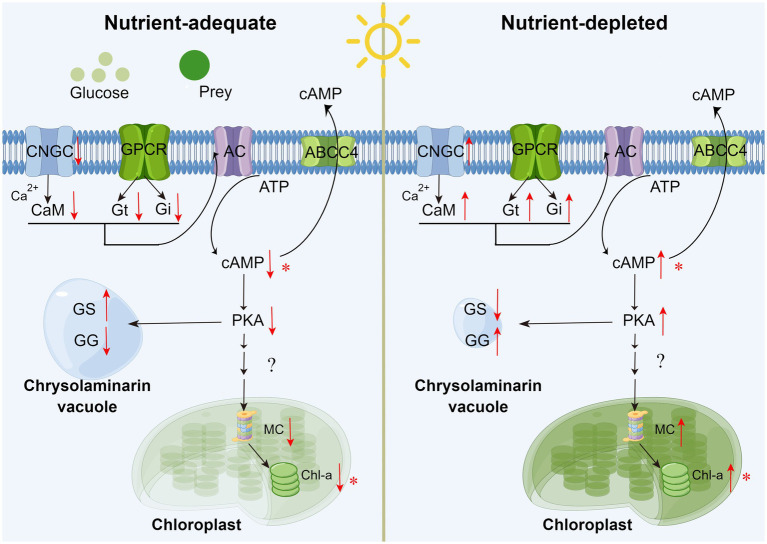
Schematic of external organics regulating the metabolism of chrysolaminarin and chlorophyll in *P. malhamensis* cells. CNGC: cyclic nucleotide gated channel subunit beta 1; CaM: calmodulin; GPCR: G Protein-Coupled Receptors; Gi/Gt: guanine nucleotide-binding protein G(i/t) subunit alpha; AC: adenylate cyclase 1; PKA: protein kinase A; MC: magnesium chelatase; GS: β-glucan synthase; GG: glucan endo-1,3-beta-D-glucosidase. Asterisks mean that the hypothesis has been verified experimentally in *P. malhamensis*. The pathway diagram was produced using the online software Figdraw.

According to the above analysis, cAMP concentration dynamics can be considered an important regulatory signal associated with the adaptation between autotrophy and mixotrophy (shown as Figure 8). When exogenous dissolved organics or particulate prey are supplied to *P. malhamensis* (i.e., adjustment from autotrophy to mixotrophy), the synthesis of cAMP will decrease through the Ca^2+^-CAM signaling pathway and GPCRs. The decrease in intracellular cAMP content will favor the accumulation of β-1,3-glucan in *P. malhamensis* and inhibit the activation of magnesium chelatase followed by a decrease in chlorophyll through a series of signal regulation pathways. After the exogenous organics have been consumed (i.e., adjustment from mixotrophy to autotrophy), β-1,3-glucan will be degraded, whereas chlorophyll will be synthesized, both as a result of increasing the intracellular cAMP content. Certainly, this pathway has many points that need to be verified in future studies, but this preliminary version nonetheless provides a possible explanation of the signal regulation that takes place during the adjustment between autotrophy and mixotrophy.

## 5. Conclusion

The present study examined the transcriptional regulation of mixotrophic *P. malhamensis* in response to different nutritional conditions, including autotrophy (P), being fed with digestible microalgae (PDA), and being fed with indigestible microalgae (PIA). Comparative analysis of RNA-seq data showed that 6,583 and 3,510 genes in *P. malhamensis* were significantly and differentially expressed in the PDA and PIA treatment, respectively, as compared to P. A global change in the cellular metabolic pathways of *P. malhamensis* when transforming from autotrophy to mixotrophy was established according to GO and KEGG enrichment results. The inhibition effect on *P. malhamensis* after grazing indigestible prey may result from the up-regulation of genes related to apoptosis and necrosis. Furthermore, the possible regulation pathway of nutritional adaptation in *P. malhamensis* mediated by the cAMP signaling molecule was preliminarily established. This study provides an improved understanding of the transcriptomic responses of mixotrophic flagellates to different nutritional conditions and thus strongly enriches the level of theoretical knowledge toward a mechanistic understanding of nutritional adaptation.

## Data availability statement

The data presented in the study are deposited in the NCBI’s Bioproject repository, accession number PRJNA934127.

## Author contributions

MM: funding acquisition, conceptualization, experiment, and writing – original draft. WY: data curation and visualization. HC and WK: experiment and writing. YG and QH: funding acquisition, writing – review and editing, and supervision – experiment design. All authors contributed to the article and approved the submitted version.
