# Supplementary material for: Transcriptional profile reveals the physiological responses to prey availability in the mixotrophic chrysophyte Poterioochromonas malhamensis
Source: Front Microbiol. 2023 Oct 4;14:1173541. doi: 10.3389/fmicb.2023.1173541 (PMC10582637; doi:10.3389/fmicb.2023.1173541)
Supplement: Supplementary file 1 [file Table_1.DOCX]

Table S1. Summary of RNA-Seq reads of *P. malhamensis* and their matches to the reference genome of *P. malhamensis*

| Sample | Replicate | Clean reads pairs | Read length | Q_30_ | Uniquely mapped (%) | Multiple mapped (%) | Total mapped (%) | Transcripts (FPKM ≥ 1) |
| --- | --- | --- | --- | --- | --- | --- | --- | --- |
| P | 1 | 40206922 | 150 | 0.95 | 30099960 (74.86) | 6913424 (17.19) | 37013384 (92.06) | 16584 |
|  | 2 | 57854520 | 150 | 0.95 | 43444946 (75.09) | 10235889 (17.69) | 53680835 (92.79) | 16596 |
|  | 3 | 41772476 | 150 | 0.95 | 30765983 (73.65) | 6982700 (16.72) | 37748683 (90.37) | 16529 |
| PDA | 1 | 38655924 | 150 | 0.95 | 40628928 (79.00) | 9499740 (18.47) | 50128668 (97.48) | 16120 |
|  | 2 | 38048756 | 150 | 0.95 | 39651701 (79.23) | 9128998 (18.24) | 48780699 (97.47) | 18007 |
|  | 3 | 49506458 | 150 | 0.95 | 29394123 (78.89) | 6692945 (17.96) | 36087068 (96.85) | 16677 |
| PIA | 1 | 51426184 | 150 | 0.94 | 30314467 (78.42) | 7050580 (18.24) | 37365047 (96.66) | 16377 |
|  | 2 | 50048606 | 150 | 0.95 | 29822943 (78.38) | 6900678 (18.14) | 36723621 (96.52) | 16708 |
|  | 3 | 37258890 | 150 | 0.95 | 38854349 (78.48) | 9026403 (18.23) | 47880752 (96.72) | 16662 |
