# Supplementary material for: Transcriptional profile reveals the physiological responses to prey availability in the mixotrophic chrysophyte Poterioochromonas malhamensis
Source: Front Microbiol. 2023 Oct 4;14:1173541. doi: 10.3389/fmicb.2023.1173541 (PMC10582637; doi:10.3389/fmicb.2023.1173541)
Supplement: Supplementary file 10 [file Presentation_1.PPTX]

## Slide 1
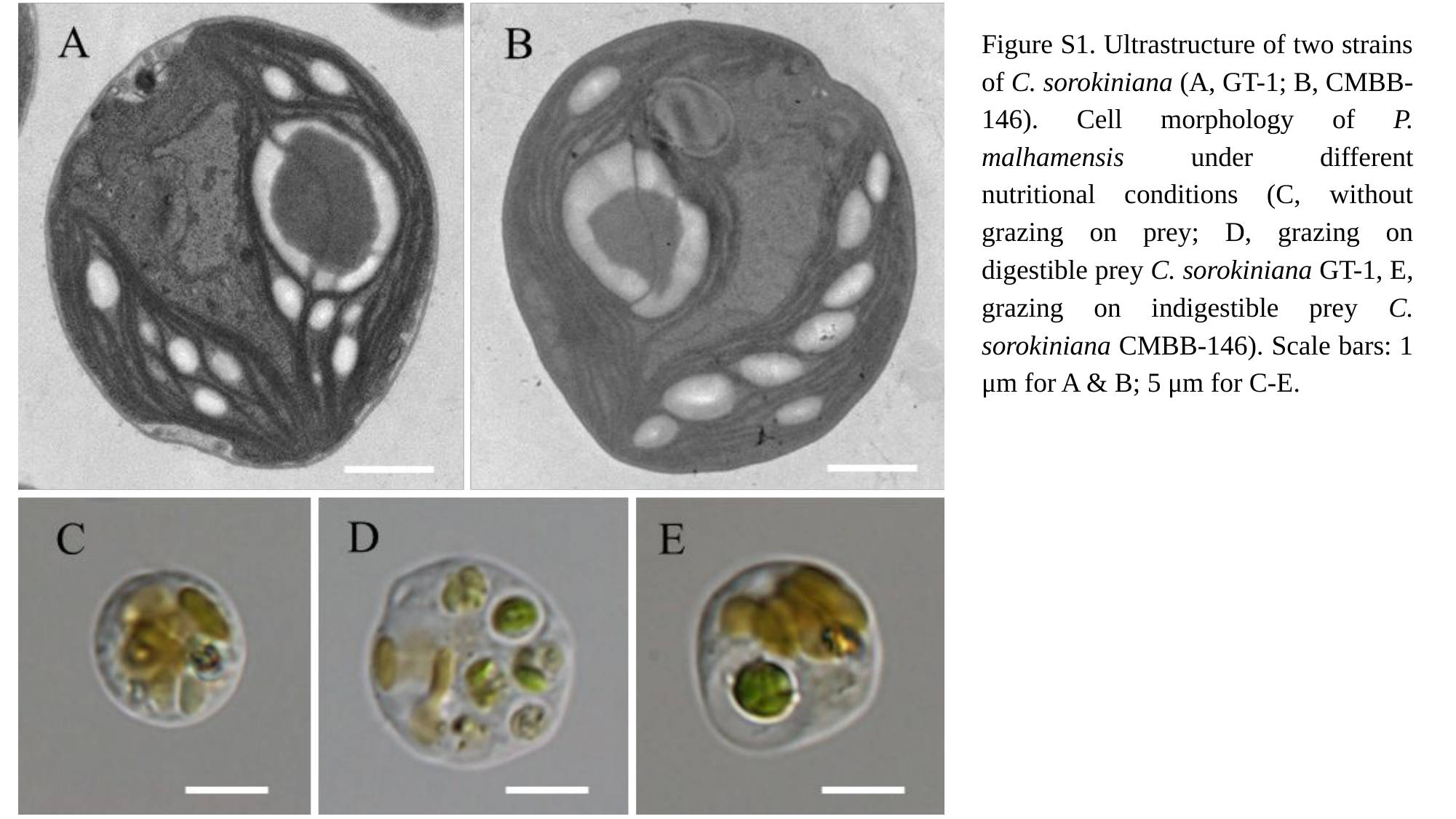

Figure S1. Ultrastructure of two strains of C. sorokiniana (A, GT-1; B, CMBB-146). Cell morphology of P. malhamensis under different nutritional conditions (C, without grazing on prey; D, grazing on digestible prey C. sorokiniana GT-1, E, grazing on indigestible prey C. sorokiniana CMBB-146). Scale bars: 1 μm for A & B; 5 μm for C-E.
